# Molecular magnetic resonance imaging

**DOI:** 10.2349/biij.2.2.e8

**Published:** 2006-04-01

**Authors:** A Hengerer, J Grimm

**Affiliations:** 1 Siemens AG, Medical Solutions, Erlangen, Germany; 2 Center for Molecular Imaging Research, MGH and Harvard Medical School, Boston, United States

**Keywords:** Molecular imaging, MRI, contrast agents

## Abstract

Molecular MRI (mMRI) is a special implementation of Molecular Imaging for the non-invasive visualisation of biological processes at the cellular and molecular level. More specifically, mMRI comprises the contrast agent-mediated alteration of tissue relaxation times for the detection and localisation of molecular disease markers (such as cell surface receptors, enzymes or signaling molecules), cells (e.g. lymphocytes, stem cells) or therapeutic drugs (e.g. liposomes, viral particles). MRI yields topographical, anatomical maps; functional MRI (fMRI) provides rendering of physiologic functions and magnetic resonance spectroscopy (MRS) reveals the distribution patterns of some specific metabolites. mMRI provides an additional level of information at the molecular or cellular level, thus extending MRI further beyond the anatomical and physiological level. These advances brought by mMRI are mandatory for MRI to be competitive in the age of molecular medicine. mMRI is already today increasingly used for research purposes, e.g. to facilitate the examination of cell migration, angiogenesis, apoptosis or gene expression in living organisms. In medical diagnostics, mMRI will pave the way toward a significant improvement in early detection of disease, therapy planning or monitoring of outcome and will therefore bring significant improvement in the medical treatment for patients.

In general, Molecular Imaging demands high sensitivity equipment, capable of quantitative measurements to detect probes that interact with targets at the pico- or nanomolar level. The challenge to detect such sparse targets can be exemplified with cell surface receptors, a common target for molecular imaging. At high expression levels (bigger than 106 per cell) the receptor concentration is approx. 10^15^ per ml, i.e. the concentration is in the micromole range. Many targets, however, are expressed in even considerably lower concentrations. Therefore the most sensitive modalities, namely nuclear imaging (PET and SPECT) have always been at the forefront of Molecular Imaging, and many nuclear probes in clinical use today are already designed to detect molecular mechanisms (such as FDG, detecting high glucose metabolism). In recent years however, Molecular Imaging has commanded attention from beyond the field of nuclear medicine. Further imaging modalities to be considered for molecular imaging primarily include optical imaging, MRI and ultrasound.

## CONTRAST AGENTS FOR mMRI

Clinical MRI scanners offer a spatial resolution of 250 µm in-plane (small bore experimental systems allow for 50 µm isotropic voxels for *in vivo* measurements), unlimited depth penetration along with exceptionally good soft tissue contrast. The above-mentioned concentration of molecular imaging targets in the micromolar range is challenging and requires sophisticated imaging strategies. Improvements in MRI design to reduce the lower detection limit are possible only to a certain extent; hence biophysical amplification mechanisms to enhance the signal from the label are necessary.

For MRI, two different classes of contrast agents exist: agents that influence mainly the signal in T2- (negative contrast agents, reducing the signal) or in T1-weighted images (positive contrast agents, increasing the signal). For both classes, methods for signal amplification have been developed. In general, both take advantage of either very high relaxivity probes, background reduction (SNR optimisation) via activation of low-relaxivity probes by the targeted molecular marker (induced changes in relaxivity) or pronounced tissue accumulation. The latter is possible only with a very restricted number of highly expressed molecular marker (e.g. fibrin for thrombosis imaging).

## Negative Contrast Agents

For T2-contrast agents, the most prominent labels are iron-oxide nanoparticles (Superparamagnetic Iron Oxide (SPIO), Very Small Paramagnetic Iron Oxide (VSPIO) or Ultrasmall Superparamagnetic Iron Oxide (USPIO)). These particles usually consist of a crystalline iron-oxide core, surrounded by polymer coating, often dextran, polyethyleneglycol or citrate. The advantage of these preparations is that each particle contains thousands of iron atoms resulting in very high T2 relaxivities of up to 200 (mMs)^-1^ [1], which makes detection of even low concentrations of contrast agents (µmol to nmol range) possible. Noteworthy, particles smaller than 300 nm also produce a substantial T1 relaxation.

Specific population of cells can be labeled with magnetic nanoparticles and followed *in vivo* (cell tracking). Using this method, the spatial distribution of immuno-competent cells into tumours over time can be studied as well as the movement of stem cells, neuronal cells or blood stem cells *in vivo* [2]. A widely excepted protocol for cell labelling using SPIO and Polyamines has been published by Frank *et al* [3]. Key concerns with cell tracking are viability, differentiability, chromosomal stability of the labelled cells as well as the stability of label affiliation with the cell.

**Figure 1:**
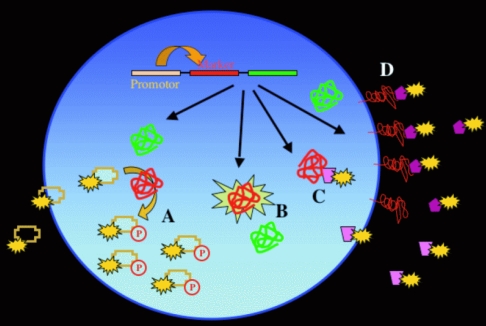
Principle of reporter genes for gene expression. The reporter gene (red) is co-expressed with the gene of interest (green), often by using the same promoter for both genes. The reporter gene can be the HSV thymidine kinase (phosphorylating radioactive-labeled nucleoside-analog, which get trapped in the cell (A), a fluorescent protein like GFP (B), an intracellular target for a marker (C) or a cell membrane receptor as the transferin-receptor (D).

Similar to PET-imaging, MRI can be used to image gene expression and to assess the efficiency of gene delivery. Viral or non-viral gene therapy schemes require targeting of the gene therapy vector to the tissue or cells of interest. Delivery of a therapeutic gene can be monitored by imaging of a reporter gene, which is introduced into the gene therapy vector and expressed together with the therapeutic gene (Figure 1). The reporter gene can be encoded for the transferrin-receptor (accumulating iron within the cell) or for tyrosinase (catalysing the synthesis of melanin) [4]. Melanin has a high iron binding capacity, which results in a high signal intensity of the henceforth melanin containing cells in T1-weighted images [5]. Compartmentalisation of iron oxide particles within targeted cells can produce significant signal amplification as well. Taking advantage of cell uptake processes via endocytosis or phagocytosis clearly exceeds the concentration that can be achieved using cell surface receptor targeting alone.

**Figure 2:**
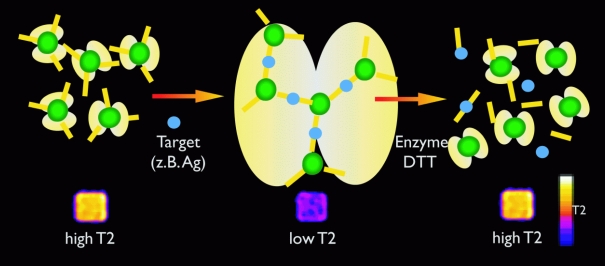
Principle of the magnetic relaxation switch (see text).

A method named “magnetic relaxation switch” facilitates the reversible activation of MR probes (Figure 2). Target-mediated aggregation of iron-oxide particles to clusters produces a significant change in the relaxivity, which can be utilised to image enzyme activities (telomerase, endonucleases, various proteinases) [6]. This cluster-formation leads to an amplification of the signal. Based on “magnetic relaxation switches”, MRI can also be used as an *in vitro* method for high throughput evaluation of biological specimens.

### Positive Contrast Agents

**Figure 3:**
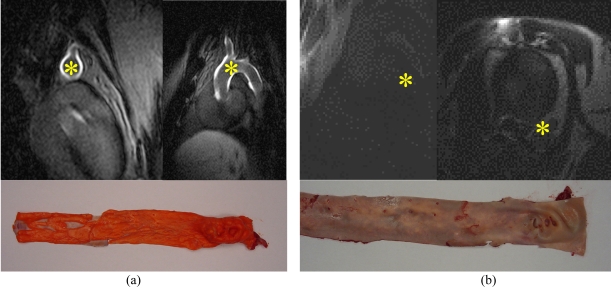
Plaque Imaging with Gadofluorine M (100 µmol/kg bw), 24 h p.i. imaged with Siemens, 1.5 T, IR turbo flash (300/4/150/20°); (a) WHHL rabbit (with plaques), (b) New Zealand White (without plaques). Asterisk (*) indicates aorta (courtesy of B. Misselwitz, Schering AG, Berlin, Germany).

Typical T1-contrast agents are small molecular weight compounds containing a single Lanthanide chelate as contrast producing element (e.g. Gadolinium-DTPA). The tissue concentration necessary to image with these T1-contrast agents on a molecular level is considerably higher than the required concentration of iron-oxide particles, it has to be in the order of mMols, since T1 relaxivity values are usually only in the in the 5-80 (mMs)^-1^ range [1]. Increasing the molecular weight can be used for enhancing the relaxivity by restricting free rotation of the Gadolinium. This can be achieved by using polymeric backbones or *in vivo* binding of small sized contrast agents to serum proteins [7]. Multilabelled macromolecular T1-contrast agents for mMRI take advantage of the fact that relaxivity is a linear function of the number of lanthanide ions per contrast agent (Figure 3) [1]. Various labelling concepts, including Gadolinium-loaded liposomes or polymeres with thousands of Gadolinium atoms, have been developed to overcome this limitation in sensitivity. For instance, the detection of angiogenetic endothelium was achieved by large Gadolinium-loaded liposomes, targeted to avb3 integrin receptors via peptides or antibodies [8]. Advantages on the MRI properties may be counterbalanced by drawbacks in pharmacokinetics and bio-availability. High molecular weight compounds have a slower diffusion rate, which restricts delivery to certain tissues such as necrotic tumour centres or the CNS.

Similar to T2 contrast agents, T1-contrast probes can be activated by different mechanisms, thus increasing the relaxivity of the agent. This target-mediated increase of relaxivity leads to an increased contrast in the presence of the target. An enzyme-mediated polymerisation (e.g. bymyeloperoxidases up-regulated in vulnerable plaques) of paramagnetic substrates into oligomers represents one possible approach. In contrast to the small monomeric substrates these polymers have a larger hydrodynamic diameter and slower rotational rate of paramagnetic metal chelates. This results in a higher longitudinal relaxivity, and since the oligomeres are eliminated much slower than the monomers all signal will come after a certain time only from the oligomere, retained at the side of the target's presence [9]. Another method utilises conformational changes in the chelates, allowing access of bulk water to the inner sphere of the chelated lanthanide upon cleavage of a protective group by an enzyme or binding of calcium to the chelate. Monitoring of gene expression with T1 contrast agents can be achieved with a carbohydrate-modified Gadolinium chelate to image the activity of the enzyme ß-galactosidase as marker gene. The enzyme cleaves the carbohydrate residue from the chelate, inducing an increase in relaxivity by allowing access of water to the gadolinium [10].

Chemical-exchange saturation transfer (CEST) agents are another emerging class of negative MRI contrast agents, which facilitate an activatable contrast. The concept of CEST can be utilised to obtain highly amplified targeted agents. The principle is based on the fact that many molecules in the body exchange protons with bulk water through the selective RF irradiation of exchanging protons. After irradiating a millimolar pool of metabolite the energy is transferred to a nearby pool of water, producing a strong increase in enhancement by means of chemical exchange. With CEST and its newer development paraCEST specific metabolites can be detected. Using this method, the concentration of metabolites such as glucose can be evaluated non-invasively *in vivo* in all organs. It furthermore allows for the measurement of the pH or the temperature. Multiparametric mMRI might become feasible as CEST agents with different absorption frequencies of the exchanging protons can be designed. The sensitivity of CEST can be improved further by incorporating larger number of exchangeable protons into a (polymeric) CEST agent. This has been accomplished by LIPOCEST agents, liposomes containing a paramagnetic shift reagent for water protons in their aqueous inner cavity [11].

Finally an “old friend” in MRI is attracting increasing attention in the course of Molecular Imaging: Fluorine-19. Xeno-labels, i.e. elements with low physiological concentration in situ but appropriate gyro-magnetic properties proved to provide superb traceability for mMRI.

## TRANSLATIONAL mMRI

To date molecular imaging is increasingly used in laboratory studies. High resolution MRI is well suited to screen mouse models for tumours and other abnormalities and can be applied to non-invasively follow up new experimental treatment strategies. While dedicated animal systems are available, clinical scanners are also very capable of obtaining high-resolution images. To further speed up translational research, a small animal MR scanner based on a clinical console has been introduced just recently (ClinScan, a joint Bruker and Siemens development). This system comes with an adapted clinical user interface and supports therefore most of the features and sequences used on clinical scanners. Standardisation of the system ensures easy protocol transfer from and to clinical scanners. Applications being developed for humans on clinical scanners will be available on ClinScan. On the other hand, any application being developed for animals can be easily transferred to human diagnostics. In addition, adaptations for clinical scanner are underway for experimental molecular imaging. These include, but are not limited to, implementing dedicated small animal protocols, (quantitative) analysis tools or dedicated small animal RF coils. In conclusion, MRI is increasingly suited to carry molecular imaging from animal models into clinical practice.

When will molecular imaging enter clinical medicine? Hot (hyper-intense) spots, which add information on the molecular mechanisms underlying a disease could guide radiologist and would be highly beneficial. Hyper-intense hot spots can be caused by highly specific accumulation of paramagnetic contrast agents. Most macromolecular Gadolinium-chelates are in an early pre-clinical research stages and the time needed to reach clinical applicability is considerable. Furthermore, toxicity concerns due to the prolonged retention of heavily Gadolinium-loaded molecules within the organism must be disproved. Superparamagnetic contrast agents can give easily detectable positive signal in combination with imaging techniques such as off resonance imaging.

Superparamagnetic iron-oxide based contrast agents are easily detectable on T2-weighted sequences, even better so on T2*-weighted images due to susceptibility effects. Iron-oxide particles currently in clinical use can be utilised for cellular imaging of phagocyting cells, if combined with peptides, helping to traverse the membrane; this applicability can be even expanded to any other cell type. Iron-oxide particles can furthermore be functionalised with a wide variety of biological active molecules such as targeting moieties of peptides, antibody (derivatives), nucleic acids or aptamers to increase specific binding. Therefore a wide variety of imaging agents for mMRI is emerging and can facilitate steps towards clinical mMRI. Translational research with iron-oxide particles will take advantage of a growing basis of installed high field MRI scanners, since the lower detection limit decreases with increasing field strength.

## DUAL MODALITY DEVICES FOR MOLECULAR IMAGING

Besides being a powerful tool for the acquisition of molecular information, MRI is capable to add anatomical or functional information (fMRI, diffusion, perfusion) to molecular images acquired with other modalities such as PET, SPECT or optical imaging. Combinations of CT and PET are increasingly used in clinical diagnostics. But there are some unique advantages for MR PET. Isocentric and simultaneous measurements are possible, no re-positioning of the patient, MRI can provide navigator technique and may give attenuation correction for PET, there is a better soft tissue contrast, and lower radiation exposure enables follow up studies. The ongoing development in imaging software and computing hardware facilitates co-registration and image fusion of physically separated units without delay. But repositioning of the patient and time interval between the scans makes fusion of separately obtained images difficult and inherently imprecise. Integrated devices would clearly be preferable even for economic reasons. Simon Cherry pioneered MR PET integration.

**Figure 4:**
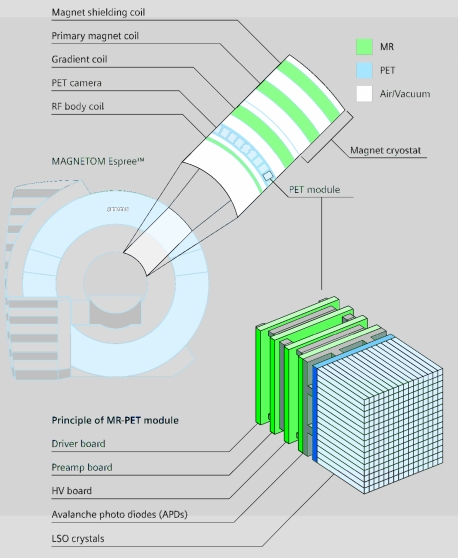
Mr-PET integrated solution. PET is acquired with a ring inserted into the magnet – simultaneous acquisition is possible.

Just recently the first fully integrated experimental set-up (based on semiconductor detectors) for MR PET has been presented by Siemens (Figure 4). Academic sites build a MR optical device. Even dual probes for optical and MR imaging have been developed [12] and triple modality probes are currently explored.

## THE BIG PICTURE

**Figure 5:**
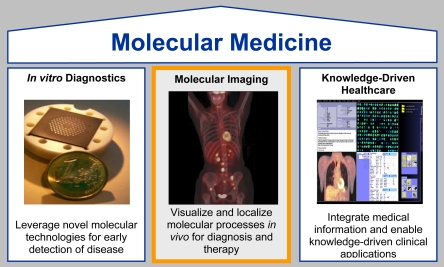
Molecular Imaging is one out of three pillars of molecular medicine.

Molecular Imaging is one out of three pillars of molecular medicine, i.e. the translation of basic molecular biology into medicine. The other two are *in vitro* diagnostics (IVD) and knowledge driven healthcare (Figure 5). To determine genetic predisposition, *in vitro* genomic or proteomic screening procedures will be used, such as DNA-chip technologies or mass spectroscopy. IT tools will be mandatory as complex molecular information, be it *in vivo* or *in vitro* diagnostic, has to be integrated by IT and has to be supplemented by knowledge bases to leverage it into clinical applications. This means diagnostic data has to be converted into meaningful medical knowledge.

The financial feasibility and medical practicability of molecular medicine including comprehensive diagnostics are debatable. The trial and error method will certainly continue to be the most reasonable approach for cost-effective therapies without side effects. However, this is not the case with highly potential therapy regimes (i.e. therapies with a potential for serious side effects), cost-intensive (molecular) treatment schemata (such as cell- or gene therapy) or therapies of chronic diseases. In such instances, a rational therapy selection based on comprehensive diagnostic data is a decisive factor for efficient and cost sensitive patient care. One of the main cost drivers in medicine is inappropriate treatment over a prolonged time. From a medical point of view, many arising molecular therapy concepts are based on individualised drugs. These treatments must be tailored to the individual biochemical set-up or disease stage of each respective patient with the support of diagnostic data. Equipped with these patient-specific data, a therapy regime is selected, taking into account the different molecular defects for each disease as well as the particular clinical history and condition of a patient.

An example of how mMRI could contribute to this scope is radiation treatment planning by semi-automated lymph nodal cancer staging using a nanoparticle-enhanced lymphotropic mMRI. Preclinical data proves that mMRI demonstrate great promise for improving quality of diagnosis in general for oncologic- (tumour angiogenesis imaging), cardiovascular- (vulnerable plaque imaging) and neurological (Alzheimer’s plaque imaging) diseases.
